# DNA Methylation Dynamics in Development and Disease: Insights from Zebrafish Models

**DOI:** 10.3390/biomedicines14051034

**Published:** 2026-05-01

**Authors:** Gan-Qiang Lai, Yan Yan, Mohini Sengupta, Ting-Hai Xu

**Affiliations:** 1 Edward A. Doisy Department of Biochemistry and Molecular Biology, Saint Louis University School of Medicine, St. Louis, MO 63104, USA; ganqiang.lai@health.slu.edu (G.-Q.L.); yan.yan@health.slu.edu (Y.Y.); 2 Department of Biology, Saint Louis University, St. Louis, MO 63103, USA; mohini.sengupta@slu.edu

**Keywords:** DNA methylation, zebrafish, DNA methyltransferase, disease model, drug discovery

## Abstract

DNA methylation is a fundamental epigenetic modification that regulates gene expression, genome stability, and cell identity across vertebrate development. Disruption of DNA methylation homeostasis contributes to a wide spectrum of human diseases, including developmental disorders, neurological conditions, and cancer. Understanding how DNA methylation patterns are established, maintained, and dynamically remodeled during development is therefore essential for elucidating disease mechanisms and identifying therapeutic opportunities. The zebrafish (*Danio rerio*) has emerged as a powerful vertebrate model for investigating DNA methylation dynamics in vivo. Its external fertilization, optical transparency, rapid embryogenesis, and high fecundity enable direct observation and experimental manipulation of epigenetic processes at developmental stages that are difficult to access in mammalian systems. In addition, the core enzymatic machinery governing DNA methylation, including DNA methyltransferase (DNMT) and ten-eleven translocation (TET) protein families, is evolutionarily conserved between zebrafish and humans. In this review, we summarize current knowledge of the zebrafish methylome and the enzymatic regulators that control DNA methylation dynamics. We discuss how DNA methylation shapes early embryonic development, organogenesis, and cell fate decisions, and highlight insights gained from zebrafish models of human disease. Finally, we examine emerging technologies that are enabling increasingly precise interrogation of epigenetic regulation in vivo. Together, these advances position zebrafish as an important platform for bridging developmental epigenetics with human disease biology and therapeutic discovery.

## 1. Introduction

**Figure 1:**
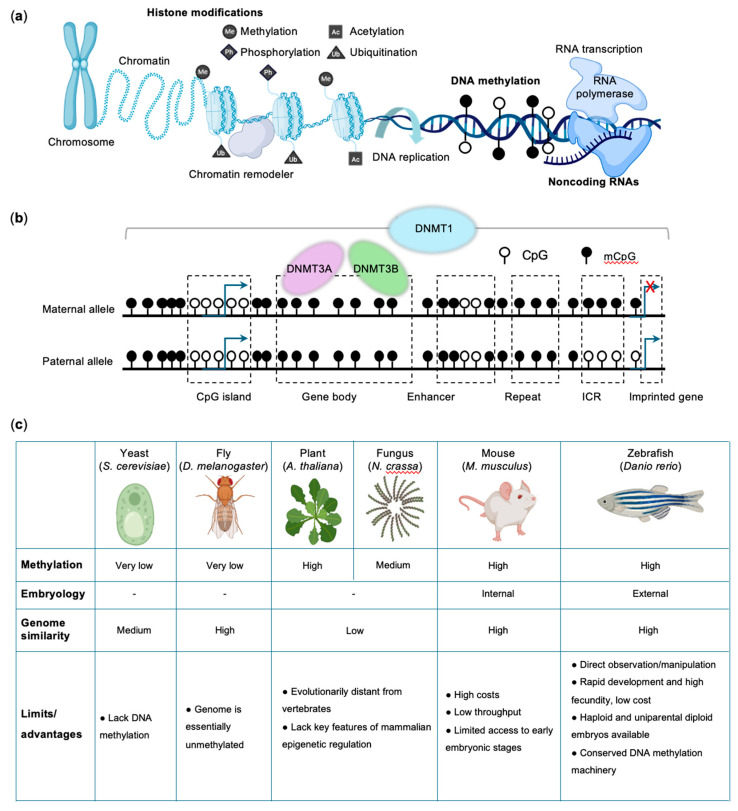
DNA methylation systems and model organisms. (**a**) Overview of epigenetic regulation. Epigenetic mechanisms, including histone modifications (methylation, acetylation, phosphorylation, and ubiquitination), DNA methylation, chromatin remodeling, and noncoding RNAs, dynamically regulate gene expression. (**b**) DNA methylation in vertebrates occurs primarily at CpG dinucleotides and is catalyzed by DNA methyltransferases (adapted from Ref. [3], Created in BioRender. Lai, G. (2026) https://BioRender.com/klhjs4, Accessed on 28 April 2026). DNMT1 maintains DNA methylation during replication, whereas DNMT3A and DNMT3B mediate *de novo* methylation. Although the majority of CpG sites in vertebrate genomes are methylated, promoter-associated CpG-rich regions, known as CpG islands (CGIs), are generally protected from methylation. Active enhancers are typically associated with low DNA methylation levels. Imprinted genes exhibit allele-specific DNA methylation at imprinting control regions (ICRs), resulting in monoallelic expression. (**c**) Comparison of methylation and genomic similarity (to human), embryology, and the advantages and limitations of model organisms in DNA methylation research.

DNA sequence-encoded genetic information is transmitted from parents to offspring and forms the foundation of classical Mendelian inheritance [1]. However, many heritable phenotypic features and human diseases cannot be fully explained by DNA sequence variation alone. Beyond the genome sequence, epigenetic information, including DNA methylation, histone modifications, chromatin remodeling, and non-coding RNAs, determines how the genetic code is interpreted in space and time (Figure 1a) [2]. These processes enable cells with identical genomes to adopt and maintain distinct functional identities and, in some contexts, allow regulatory states to be transmitted across cell divisions or even generations [3,4]. Epigenetic regulation is dynamically established during development and in response to environmental cues, and its dysregulation has been increasingly implicated in human developmental disorders and diseases [5].

Among epigenetic mechanisms, DNA methylation represents one of the most stable and evolutionarily conserved regulatory modifications in vertebrates [6]. It primarily occurs through the covalent addition of a methyl group to the 5-position of cytosine, generating 5-methylcytosine (5mC), predominantly within CpG dinucleotides [7]. DNA methylation patterns are established by the *de novo* DNA methyltransferases (DNMT) 3A and 3B [8,9], and faithfully maintained during cell divisions by DNMT1 (Figure 1b) [10,11]. In addition to canonical CpG methylation, non-CpG methylation has been observed in specific developmental contexts, particularly in pluripotent cells and neurons, suggesting additional regulatory complexity [12]. Proper establishment and maintenance of DNA methylation are therefore essential for regulating gene expression, maintaining genomic stability, and preserving cellular identity. Consistent with these roles, disruption of DNA methylation has been linked to a broad spectrum of human diseases, including congenital developmental disorders, neurodevelopmental syndromes, cancer, and age-related pathologies [13].

Model organisms have played a key role in advancing our understanding of DNA methylation. However, several classical model systems, including *Saccharomyces cerevisiae*, *Drosophila melanogaster*, and *Caenorhabditis elegans*, lack detectable DNA methylation [14]. Consequently, many foundational discoveries have relied on alternative systems such as the mouse, the plant *Arabidopsis thaliana*, and the fungus *Neurospora crassa* [15]. Although these systems offer important advantages, each also presents limitations for understanding mammalian epigenetic regulation. Rodent models, for example, provide physiological relevance but are constrained by high costs, lower experimental throughput, and limited access to early embryonic stages when extensive epigenetic reprogramming occurs [16]. By contrast, plant and fungal models are genetically tractable but evolutionarily distant from vertebrates and lack key features of mammalian epigenetic regulation, limiting their direct relevance to human disease (Figure 1c).

The zebrafish (*Danio rerio*) has emerged as a powerful complementary vertebrate model that overcomes many of these limitations [17]. Approximately 70% of human genes have at least one zebrafish ortholog [18], and comparative methylome analyses indicate that the zebrafish DNA methylation system shares substantial similarity with that of mammals [19]. Zebrafish embryos develop externally and are optically transparent, enabling direct observation and experimental manipulation from the single-cell stage onward. Their rapid development and high fecundity support statistically robust experiments and large-scale genetic or chemical screens [20]. In addition, haploid and uniparental diploid embryos can be readily generated, facilitating precise dissection of maternal and paternal contributions to the methylome [21]. Zebrafish are also highly amenable to forward genetic screens, genome-editing approaches, and high-throughput drug screening, enabling systematic identification of regulators of epigenetic pathways while facilitating both disease modeling and therapeutic discovery [22]. Importantly, zebrafish retain a conserved DNA methylation machinery, including homologs of DNA methyltransferase (DNMT) and ten-eleven translocation (TET) proteins, while also exhibiting distinctive epigenetic features during early development (Figure 1c). For example, unlike mammals, zebrafish development does not rely on genomic imprinting for viability, making them a simplified vertebrate system for dissecting the functions of DNA methylation during development [3].

In this review, we summarize current knowledge of DNA methylation dynamics with particular focus on insights from the zebrafish model. We first outline the global features of zebrafish methylomes and compare them with those of mammals. We then discuss the enzymatic machinery responsible for establishing and modifying DNA methylation, including DNMT and TET proteins. Next, we discuss how DNA methylation is remodeled during early embryogenesis and how it contributes to lineage specification and organogenesis. We further highlight how zebrafish models have advanced our understanding of DNA methylation-associated human diseases. Finally, we discuss emerging technologies that are accelerating epigenetic discovery and shaping future directions in the field.

## 2. The DNA Methylation Landscape in Zebrafish

Genome-wide profiling has revealed that the zebrafish methylome shares many fundamental architectural features with those of other vertebrates while exhibiting distinctive developmental dynamics. In vertebrate genomes, DNA methylation occurs primarily at CpG dinucleotides and constitutes one of the most widespread epigenetic modifications (Figure 1b) [15]. Approximately 70–80% of CpG sites are methylated in zebrafish, mice, and humans, resulting in broadly distributed genome-wide methylation with defined regional exceptions. The major exception consists of CpG-rich regions known as CpG islands (CGIs), which are commonly located at gene promoters and frequently overlap transcription start sites [23]. Promoter-associated CGIs are typically protected from methylation in actively transcribed genes, whereas hypermethylation of these regions is associated with transcriptional silencing through reduced transcription factor accessibility and the recruitment of methyl-CpG-binding proteins [24].

Beyond promoter regions, CpG methylation is widely distributed across intergenic sequences, repetitive elements, and gene bodies (Figure 1b), where it contributes to suppression of inappropriate transcriptional initiation, repression of transposable elements, and transcriptional stability, thereby supporting genome integrity [6,25]. Notably, zebrafish genome contains a relatively high proportion of repetitive and intergenic sequences, including abundant transposable elements, which contribute to the overall elevated levels of DNA methylation compared with mammals. This feature likely reflects the increased requirement for methylation-based repression of transposable elements [26]. In addition, DNA demethylation at enhancer regions in zebrafish is positively correlated with transcriptional activation of adjacent genes, supporting an important role for methylation dynamics at distal regulatory elements [20]. Comparative analyses of DNA methylation-dependent gene regulation in zebrafish and mammals, therefore, provide valuable insights into the evolutionary diversification of vertebrate gene regulatory mechanisms.

Although CpG methylation constitutes the dominant form of DNA methylation in vertebrates, additional sequence contexts contribute to methylome complexity. In contrast to plants and fungi, which exhibit substantial non-CpG methylation in CpHpG and CpHpH contexts (H = A, C, or T), non-CpG methylation in vertebrates is comparatively limited [27]. In mammals, CpHpG and CpHpH methylation is largely restricted to pluripotent stem cells and specific differentiated lineages such as neurons and it generally occurs at substantially lower levels than CpG methylation [12]. Zebrafish display similarly low levels of non-CpG methylation. However, emerging evidence indicates that these modifications can be developmentally regulated and enriched in specific cell types, suggesting conserved regulatory roles in particular biological contexts [23]. While the functional significance of non-CpG methylation in zebrafish remains less well characterized than in mammals, its presence underscores the complexity of vertebrate methylomes and suggests that multiple forms of DNA methylation contribute to fine-tuning gene regulation during development. Together, these features define a conserved vertebrate methylation architecture characterized by globally high CpG methylation, protection of promoter CpG islands, enrichment within gene bodies and repetitive elements, and relatively minor contributions from non-CpG methylation (Figure 1c).

Despite these broadly conserved architectural features, important species-specific differences exist in methylome dynamics during early development. In mammals, early embryogenesis and germ cell development are characterized by extensive waves of genome-wide demethylation followed by *de novo* remethylation, processes that are closely linked to the establishment of totipotency and genomic imprinting [26]. By contrast, zebrafish embryos follow a markedly different strategy. Following fertilization, the paternal methylome is largely retained, whereas the maternal methylome undergoes progressive remodeling toward a sperm-like pattern prior to zygotic genome activation [20]. These differences likely reflect differences in reproductive biology, including the absence of genomic imprinting and extraembryonic tissues in zebrafish.

Nevertheless, many regulatory features of the zebrafish methylome closely resemble those observed in mammals. Core genomic characteristics, including heavy methylation of gene bodies and repetitive elements, hypomethylation of promoter CpG islands in actively transcribed genes, and dynamic methylation of developmentally regulated loci (Figure 1b), are broadly conserved across vertebrates [25]. Aberrant CpG island hypermethylation at gene promoters, a hallmark of many cancers that results in the silencing of tumor suppressor genes and developmental regulators, is also observed in zebrafish [28]. Several disease-associated epigenetic pathways are preserved, including DNMT3A- and TET-mediated regulation of hematopoiesis, DNMT1- and UHRF1-dependent maintenance methylation, and methylation-mediated repression of transposable elements [29].

At the same time, notable divergences highlight species-specific adaptations in epigenetic regulation. Mammals process imprinting-dependent differentially methylated regions (DMRs) and placental-specific epigenetic programs that are not present in zebrafish, and zebrafish also lack a clear ortholog of *DNMT3L*, indicating reduced dependence on parent-of-origin methylation control [21]. These distinctions, however, do not diminish the zebrafish’s utility as a model system. Rather, they provide a simplified and experimentally tractable platform to dissect the core principles of vertebrate DNA methylation, understand regulatory divergence, and model human disease in a vertebrate context [17].

While these global features outline the architectural organization of the zebrafish methylome, understanding how this landscape is established and dynamically regulated requires examining the enzymatic machinery responsible for DNA methylation and demethylation.

## 3. Enzymatic Machinery of DNA Methylation in Zebrafish

### 3.1. The Zebrafish dnmt Gene Repertoire

**Figure 2:**
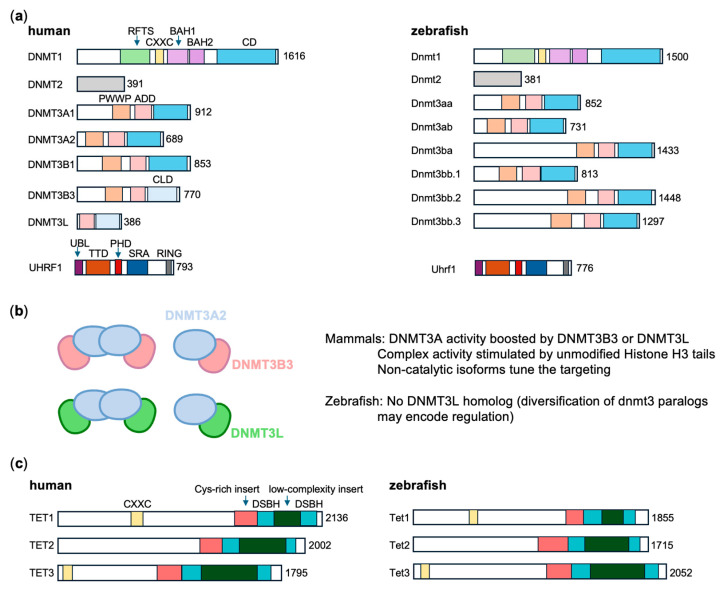
DNA methyltransferase and demethylase orthologs in zebrafish and humans. (**a**) Domain organization of DNA methyltransferases and UHRF1 orthologues. DNA methyltransferase orthologs share a conserved C-terminal methyltransferase (MTase) catalytic domain (CD) or catalytic-like domain (CLD). DNMT1, DNMT3A1, DNMT3A2, and DNMT3B1 are catalytically active and mediate the generation of 5-methylcytosine (5mC), whereas DNMT3B3 and DNMT3L lack catalytic activity. In contrast, the N-terminal regions exhibit distinct domain architectures that contribute to differential chromatin targeting and regulatory functions. RFTS, replication foci targeting sequence; CXXC, cysteine-rich domain; BAH, bromo-adjacent homology domain; PWWP, Pro–Trp–Trp–Pro domain; ADD, ATRX–DNMT3–DNMT3L domain. UHRF1 orthologs are conserved in humans and zebrafish. The ubiquitin-like (UBL) domain at the N-terminus contributes to ubiquitination activity, whereas the tandem Tudor domain (TTD) and plant homeodomain (PHD) finger mediate binding to methylated histone H3, including H3K9me2/3. The SET and RING-associated (SRA) domain supports maintenance of DNA methylation and histone modifications by recruiting DNMT1 and HDAC1, respectively. The Really Interesting New Gene (RING) domain at the C-terminus confers intrinsic E3 ubiquitin ligase activity toward histone and non-histone substrates. (**b**) Model of DNMT3 complexes assembly in mammals [31]. (**c**) Domain organization of TET demethylases. TET demethylase orthologs contain a C-terminal catalytic domain comprising a double-stranded β-helix (DSBH) with a low-complexity insert and a cysteine-rich insert region. Notably, TET1 and TET3 contain CXXC domains that bind unmethylated CpG sequences.

The establishment, maintenance, and remodeling of DNA methylation patterns in vertebrates are mediated by a conserved set of DNMTs (Figure 2a and Table 1). In mammals, DNMT3A and DNMT3B are primarily responsible for *de novo* DNA methylation [24], whereas DNMT1, acting in concert with UHRF1, is responsible for maintenance of DNA methylation [30]. Mammalian genomes also encode DNMT3L, a catalytically inactive homolog that functions as a regulatory scaffold to stimulate DNMT3A and DNMT3B activity and to couple DNA methylation to chromatin states [5,31].

Comparative sequence analysis reveals that the zebrafish genome encodes an expanded repertoire of *dnmts* (Figure 2a and Table 1), including orthologs of the conserved *DNMT1*, *DNMT2*, and *DNMT3* families in mammals [46]. Zebrafish possess a single *dnmt1* gene corresponding to mammalian *DNMT1,* as well as multiple *DNMT3*-related paralogs. These include *dnmt3aa* (*dnmt8*) and *dnmt3ab* (*dnmt6*), closely related to mammalian *DNMT3A*, and *dnmt3bb.1* (*dnmt4*), resembling mammalian *DNMT3B*. Additional paralogs, *dnmt3bb.2* (*dnmt3*), *dnmt3bb.3* (*dnmt5*), and *dnmt3ba* (*dnmt7*), represent teleost-specific expansions, likely arising from lineage-specific duplication events (Figure 2a and Table 1) [21,47]. Rather than representing simple redundancy, these paralogs display distinct temporal and spatial expression patterns, suggesting functional diversification and specialization of *de novo* methylation activities in zebrafish.

Across all *DNMT* paralogs, *DNMT2* is the most evolutionarily conserved (Figure 2a). Zebrafish harbor a single *dnmt2* gene that is widely expressed and primarily methylates tRNA^Asp^ rather than genomic DNA [48]. The presence of *dnmt1* and multiple *dnmt3* homologs in zebrafish highlights the evolutionary conservation of maintenance and *de novo* methylation mechanisms among vertebrates. While the genomic repertoire establishes the molecular toolkit, functional conservation with human DNMTs provides insight into the mechanistic principles governing DNA methylation in zebrafish.

### 3.2. Functional Correspondence to Human DNMTs Proteins

Dnmt1 was the first DNMT characterized in zebrafish and provided early insights into the evolutionary conservation of the DNA methylation machinery [49]. Zebrafish Dnmt1 shares approximately 73% overall sequence identity and nearly 89% identity within the catalytic domain with its human ortholog, retaining key regulatory domains such as the N-terminal BAH and CXXC domains (Figure 2a) [21]. Similarly, zebrafish encode a UHRF1 ortholog with about 66% sequence identity to its human counterpart, preserving critical SRA, ubiquitin-like, and PHD domains that couple DNA replication to maintenance methylation (Figure 2a) [50]. Loss of Dnmt1 in zebrafish leads to global hypomethylation, derepression of transposable elements, and severe developmental defects, underscoring its essential role in genome stability and embryogenesis [51].

Mammalian DNMT3A contains three major functional domains: the Pro-Trp-Trp-Pro (PWWP) domain, which mediates chromatin targeting by recognizing histone H3K36 methylation [52,53,54]; the ATRX-DNMT3A-DNMT3L (ADD) zinc-finger domain, which specifically interacts with the histone H3 N-terminal tail and relieves autoinhibition of DNMT3A or DNMT3B in the absence of histone H3K4 methylation [55,56,57]; and the C-terminal DNA methyltransferase (MTase) domain responsible for *de novo* catalytic activity (Figure 2a and Table 1) [58,59]. Zebrafish DNMT3 paralogs also conserve PWWP, ADD, and MTase domains, suggesting similar chromatin-targeting mechanisms [46].

Our previous structural studies have provided important mechanistic insights into how DNMT3 complexes engage chromatin (Figure 2b and Table 1). For instance, structural analysis of the nucleosome-bound DNMT3A2–DNMT3B3 complex demonstrated that DNMT3B3, as an accessory subunit, engages the nucleosome acidic patch to position the catalytically active DNMT3A2 subunit toward linker DNA, facilitating genome-wide DNA methylation [33]. In contrast, cryo-EM structures of nucleosome-bound DNMT3A2–DNMT3L complex revealed a distinct mechanism in which DNMT3L functions primarily as a histone modification sensor rather than an acidic patch anchor due to the 180° rotation of a “switching helix” in DNMT3L. Dynamic oligomerization of the DNMT3A2–DNMT3L complex suggests an additional layer of allosteric regulation governing chromatin targeting and catalytic activity [31]. Together, these studies define complementary yet mechanistically distinct modes by which DNMT3 complexes achieve regulated *de novo* DNA methylation on chromatin.

A notable difference between zebrafish and mammals is the absence of a clear *DNMT3L* ortholog in zebrafish (Figure 2a and Table 1). In mammals, DNMT3L, primarily expressed in embryonic stem cells, is essential for establishing maternal imprints [60,61]. Zebrafish do not require genomic imprinting for viability, as demonstrated by fertile androgenetic and parthenogenetic adults [62]. The absence of the *DNMT3L* ortholog in zebrafish may therefore reflect fundamental differences in imprinting architecture and epigenetic reprogramming dynamics, and the teleost-specific expansion of *DNMT3* paralogs may provide alternative regulatory mechanisms that compensate for the absence of the *DNMT3L* ortholog [63,64]. This divergence highlights the flexibility of epigenetic networks and raises questions about how conserved methylation outcomes are achieved through distinct molecular strategies.

### 3.3. DNA Demethylation by TET Proteins

Beyond establishment, dynamic regulation also requires active DNA demethylation, mediated by the TET family of dioxygenases [65]. DNA demethylation in mammals can occur through both passive and active mechanisms [66]. Passive demethylation results from replication-dependent dilution of 5mC when DNMT1 activity is reduced or inhibited. Active demethylation involves sequential oxidation of 5mC to 5-hydroxymethylcytosine (5hmC), 5-formylcytosine (5fC), and 5-carboxycytosine (5caC) by TET dioxygenases (TET1-3) [5]. Notably, all three oxidized 5mC derivatives are poorly recognized by DNMT1, thereby facilitating passive loss of methylation during DNA replication [67]. Additionally, 5fC and 5caC, but not 5hmC, can be removed from DNA by thymine DNA glycosylase (TDG) and restored to unmodified cytosine through base excision repair (BER) [68].

Zebrafish, like mammals, possess single orthologs of human *TET1*, *TET2*, and *TET3* (Figure 2b and Table 1), which similarly mediate 5mC oxidation followed by TDG-dependent repairs, contributing to active demethylation dynamics [69]. Collectively, the zebrafish genome encodes a conserved yet diversified enzymatic toolkit for regulating DNA methylation. Dnmt1 maintains methylation, multiple DNMT3 paralogs mediate *de novo* methylation, and TET-dependent pathways facilitate demethylation, together establishing the foundation for dynamic methylation landscapes during development.

## 4. DNA Methylation Dynamics During Early Embryogenesis

Early embryonic development is accompanied by extensive epigenetic remodeling that establishes the foundation for subsequent cell fate decisions [70]. Among these changes, dynamic regulation of DNA methylation plays a central role in resetting epigenetic states and enabling the transition from gametic genomes to a developmentally competent embryonic methylome [71].

**Figure 3:**
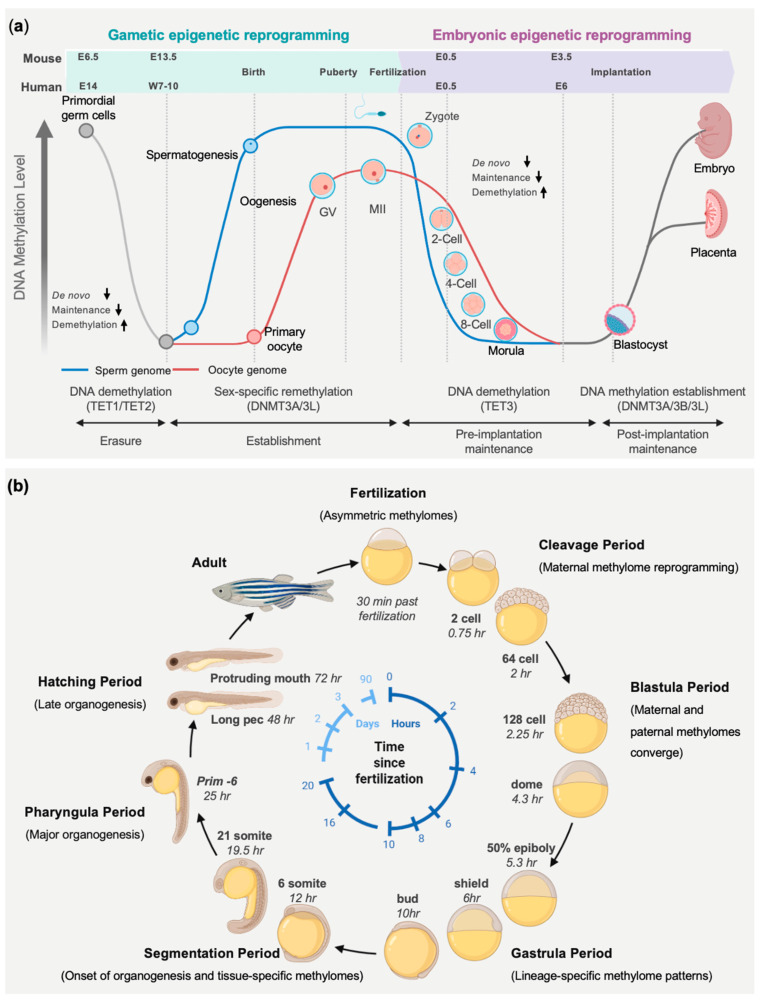
DNA methylation dynamics during early embryogenesis. (**a**) Embryonic and germline DNA methylation erasure and re-establishment (Adapted from Ref. [24], Created in BioRender. Lai, G. (2026) https://BioRender.com/clxtbdo, Accessed on 28 April 2026). Post-implantation, primordial germ cells (PGCs) undergo two waves of genome-wide DNA demethylation: a passive dilution and an active process mediated by TET1 and TET2. Sex-specific remethylation is subsequently established by DNMT3A and DNMT3L, occurring prenatally in males and during oocyte growth in females. After fertilization, TET3 drives active DNA demethylation of the parental genome. Following passive demethylation, DNA methylation levels reach a minimum at the blastocyst stage. *De novo* methylation is then re-established by DNMT3A, DNMT3B, and DNMT3L after implantation. Extraembryonic tissues, such as the placenta, remain relatively hypomethylated, consistent with their lower expression of *de novo* DNA methyltransferases. (**b**) DNA methylation dynamics during zebrafish embryogenesis (Adapted from Refs. [78,79], Created in BioRender. Lai, G. (2026) https://BioRender.com/clxtbdo, Accessed on 28 April 2026). Following fertilization, the paternal genome remains highly methylated, whereas the maternal genome exhibits a distinct methylation pattern, resulting in asymmetric parental methylomes. During cleavage (0–2 hpf), the maternal methylome undergoes progressive reprogramming toward a sperm-like state without global demethylation. By the blastula stage (~2–5.3 hpf), parental methylation methylomes converge, establishing a globally methylated embryonic genome prior to zygotic genome activation. During gastrulation (~5.3–10 hpf), lineage-specific DNA methylation patterns emerge, coinciding with germ layer formation and early cell fate specification. Organogenesis begins in the segmentation period (~10–24 hpf), where tissue-specific methylation landscapes are established. This process continues through the pharyngula period (~24–48 hpf), representing major organogenesis, during which organs differentiate and functional programs are stabilized. In the hatching/early larval period (~48–72 hpf), methylation patterns become increasingly stable and support mature organ function and maintenance of cell identity. hpf: hours post-fertilization.

In mammals, two major waves of genome-wide DNA methylation reprogramming occur (Figure 3a). The first takes place shortly after fertilization, when parental methylation patterns are extensively erased to establish totipotency [72], and the second occurs in primordial germ cells (PGCs), where methylation marks are again globally removed before sex-specific methylation patterns are re-established [73]. These reprogramming events promote embryonic plasticity and enable the establishment of germline-specific epigenetic states. In both contexts, DNA methylation erasure is mediated by both passive and active mechanisms. Following fertilization, the parental genome undergoes rapid active TET3-dependent demethylation [74,75], whereas the maternal genome is initially protected by PGC7 (also known as DPPA3 or STELLA) and largely demethylated through replication-dependent passive dilution [76,77]. Subsequent studies suggest that a fraction of the maternal methylome may also undergo active demethylation during early embryogenesis. By the blastocyst stage, global methylation reaches its lowest levels in the inner cell mass (ICM) and trophectoderm [75].

Implantation marks a critical transition during which genome-wide DNA methylation is re-established [80]. The pluripotent epiblast expands and establishes epithelial polarity [81], while *de novo* DNA methylation restores global methylation levels across the genome by DNMT3A and DNMT3B with partially distinct roles: germ cells depend mainly on DNMT3A in cooperation with DNMT3L, whereas embryonic remethylation is largely mediated by DNMT3B. Consistently, *Dnmt3b* deficiency in mice results in more pronounced post-implantation methylation defects than loss of *Dnmt3a* [82,83]. These transitions in DNA methylation landscapes coincide with large-scale chromatin reorganization, including progressive redistribution of Polycomb group proteins to DNA methylation valleys (DMVs) [84,85].

In contrast, zebrafish embryogenesis follows a fundamentally different epigenetic strategy (Figure 3b). Development occurs externally, does not rely on genomic imprinting, and proceeds without genome-wide post-fertilization DNA demethylation. Zebrafish embryos inherit a relatively stable paternal methylome, whereas the maternal methylome is progressively reprogrammed toward a sperm-like methylation state prior to biallelic zygotic transcription [63,64]. This remodeling occurs independently of the paternal template, indicating that the paternal methylome primarily serves as a reference for establishing the embryonic methylation landscape [64]. Early zebrafish embryos neither generate extraembryonic tissues nor require inductive germline specification, as primordial germ cells are predetermined by maternally inherited germ plasm [86]. Consequently, global epigenetic erasure is unnecessary [87]. Instead, the zebrafish gametic methylome is largely preset, with only limited maternal adjustments during early cleavage stages [63,64].

Genome-wide bisulfite sequencing has revealed quantitative differences between gametic methylomes. Sperm genomes display very high levels of CpG methylation (~91–95%), whereas oocytes exhibit lower levels (~75–80%) [63,64]. Non-CpG methylation is largely absent during early zebrafish development. After fertilization, global CpG methylation declines modestly, reaching a minimum around the 64-cell stage, and is then progressively restored to sperm-like levels by the sphere stage [64]. A key developmental milestone during this process is zygotic genome activation (ZGA), which occurs during the maternal-to-zygotic transition (MZT). At this stage, transcriptional control shifts from maternally deposited transcripts to the newly activated embryonic genome [34,88]. DNA methylation contributes to this transition by shaping promoter accessibility and enhancer activity, thereby establishing a chromatin environment permissive for transcriptional activation [88]. Proper coordination between methylation dynamics and transcriptional activation is essential for normal embryogenesis, and disruption of Dnmts activity during this window can lead to widespread transcriptional dysregulation and developmental defects [89,90]. By zygotic genome activation, the maternal methylome has largely converged into a unified embryonic methylation landscape. Despite the absence of global methylome erasure, selective remodeling occurs at specific regulatory regions. Most promoter CpG islands remain stable, with hypomethylated transcription start sites associated with housekeeping and early developmental genes, while hypermethylated promoters are typically enriched for genes involved in later development, such as neurogenesis [91]. A limited subset of CpG islands undergoes dynamic methylation changes during this period, including demethylation of germline and early embryonic regulators before the midblastula transition (MBT) and methylation of oogenesis-associated genes during zygotic activation [64].

Together, these findings indicate that early zebrafish embryogenesis involves selective remodeling of regulatory regions rather than global erasure of the methylome. Although the strategies differ markedly from those in mammals, both systems ultimately establish developmentally competent epigenetic landscapes that support genome stability, transcriptional activation, and lineage specification. This highlights the evolutionary flexibility of epigenetic regulation and demonstrates that conserved enzymatic machinery can operate within distinct regulatory frameworks to achieve robust vertebrate development.

## 5. DNA Methylation in Organogenesis and Cell Fate Specification

Following the establishment of a developmentally competent methylome during early embryogenesis, DNA methylation remains a central regulator of organogenesis and cell fate specification. By stabilizing lineage-specific transcriptional programs and preventing inappropriate gene activation, DNA methylation guides progenitor cells (PCs) as they progressively restrict developmental potential [92]. Zebrafish, with their optical accessibility and rapid external development, provide a powerful vertebrate system for examining how dynamic methylation landscapes orchestrate tissue differentiation.

### 5.1. DNA Methylation in Hematopoietic Development

Hematopoiesis exemplifies how DNA methylation regulates cell fate. In vertebrates, TET-mediated DNA demethylation is essential for lineage progression, with loss of *Tet1*, *Tet2*, and *Tet3* in mice disrupting differentiation, enhancing proliferation, and perturbing cytokine signaling [93,94]. TET proteins frequently interact with lineage-specific transcription factors to guide their recruitment to defined genomic loci, thereby shaping cell type-specific methylation landscapes [95]. In zebrafish, Tet2 and Tet3 function as the dominant 5mC dioxygenases during embryogenesis and are jointly required for hematopoietic stem cell (HSC) emergence via regulating Notch signaling in hemogenic endothelium. Restoration of the gata2b-scl-runx1 transcriptional cascade rescues HSC defects in Tet2/3 double mutants [96].

Zebrafish hematopoiesis occurs in three development waves: (i) the primitive wave, generating spatially segregated erythroid and myeloid lineages including neutrophils, macrophages, and mast cells; (ii) the transient wave, in which erythromyeloid progenitors (EMPs) produce erythroid and myeloid derivatives; and (iii) the definitive wave, establishing long-term HSCs around 5 days post-fertilization, which sustain multilineage hematopoiesis, including lymphoid development, throughout adulthood [97,98]. *De novo* DNA methylation is critically involved in this process. The zebrafish DNA methyltransferase Dnmt3bb.1 functions downstream of Notch1 and Runx1 to maintain hematopoietic stem and progenitor cell (HSPC) identity by promoting cmyb methylation and expression. Loss of Dnmt3b depletes HSPCs, while ectopic expression enhances hematopoietic development [40].

These mechanisms are highly conserved in humans: *DNMT3A* is among the most frequently mutated genes in acute myeloid leukemia (AML), with recurrent mutations at R882 and additional variants throughout the coding sequence [99,100]. The conservation of hematopoietic methylation programs between zebrafish and humans, therefore, positions zebrafish as a powerful in vivo model for studying DNMT3 dysfunction and leukemogenesis.

### 5.2. DNA Methylation in Neurodevelopment

DNA methylation is also essential for regulating gene expression during vertebrate neurodevelopment. DNMT3A-mediated intergenic methylation accumulates during postnatal brain development, coinciding with the progressive deposition of non-CpG methylation in postmitotic neurons [37,101]. Disruption of methylation recognition can profoundly affect neural function. For example, Rett syndrome, a monogenic neurodevelopmental disorder within the autism spectrum, is caused by loss-of-function mutations in MeCP2, a key reader of CpG methylation in neurons [102,103]. Functional studies in zebrafish further reveal tissue-specific roles for DNA methyltransferases during neural development. Knockdown of *dnmt3bb.2* leads to severe defects in the brain and retina, whereas depletion of *dnmt1* produces distinct abnormalities, indicating that these enzymes perform nonredundant roles during neural differentiation [104].

### 5.3. Organogenesis and Tissue Homeostasis

Beyond the hematopoietic and nervous systems, DNA methylation contributes broadly to organogenesis across multiple tissues [5]. During embryonic development, dynamic methylation helps establish lineage-specific transcriptional programs that guide the differentiation of progenitor cells into specialized tissues [105]. These methylation patterns reinforce appropriate gene expression states while repressing alternative lineage genes, thereby stabilizing cellular identity. In zebrafish, rapid external development and powerful genetic tools provide a uniquely accessible system for dissecting how epigenetic mechanisms shape organ formation and long-term tissue maintenance [3]. Disruption of zebrafish maintenance methyltransferase Dnmt1 leads to global hypomethylation and pronounced differentiation defects in several tissues, including the retina, intestine, and exocrine pancreas, while other organs develop relatively normally [106]. These observations highlight the tissue-specific sensitivity of developmental programs to methylation perturbation.

DNA methylation also contributes to maintaining tissue homeostasis after embryogenesis. As organs mature, stable methylation patterns preserve differentiated cellular states while allowing limited plasticity in response to physiological cues [5]. Zebrafish provide a particularly informative model for studying these processes because many tissues exhibit robust regenerative capacity. Organs such as the heart, liver, and caudal fin can regenerate efficiently following injury [107]. Genome-wide epigenomic profiling of regenerating zebrafish fins has revealed that lineage-specific DNA methylation patterns remain largely stable during regeneration, indicating that differentiated cells retain their epigenetic identity while contributing to blastema formation [108].

### 5.4. Germline Development

Germline specification represents a distinctive context in which DNA methylation contributes to developmental regulation and transgenerational inheritance (Figure 3a). Germ cells give rise to gametes and uniquely transmit both genetic and epigenetic information across generations [109]. Across animals, primordial germ cells (PGCs) arise through two principal strategies: pre-formation and induction. In experimental model organisms such as *Drosophila*, *C. elegans*, *Xenopus laevis*, and zebrafish, the germline is specified by pre-formation [110]. In this process, cells inheriting maternal RNAs and proteins form germ plasm and adopt germline identity, whereas remaining cells differentiate into somatic tissues. In zebrafish, germ plasm components such as *vasa* and *nanos* are asymmetrically distributed during early cleavage stages, leading to early segregation of PGCs from the somatic lineage [111]. This mechanism effectively protects germline identity from somatic differentiation programs and allows germ cell fate to be specified very early in development.

In contrast, mammals specify germ cells through inductive signaling [112]. Primordial germ cell induction occurs after the differentiation of the trophectoderm and primitive endoderm [113]. Germline specification is accompanied by extensive epigenomic reprogramming, including genome-wide DNA demethylation that erases parental imprints and X-chromosome inactivation marks in females [24,114]. However, the full physiological and developmental roles of these reprogramming events remain poorly understood. After fertilization, the differentially methylated parental genomes undergo a second phase of epigenetic remodeling before re-establishment during implantation. Together, these epigenetic transitions create a recurring cycle of genome-wide hypermethylation and hypomethylation that underpins changes in cell identity and developmental potential [5].

Collectively, studies in zebrafish demonstrate that DNA methylation functions as a central regulatory mechanism linking early developmental epigenetic states to lineage-specific gene expression programs. By stabilizing transcriptional networks while constraining inappropriate plasticity, DNA methylation ensures the orderly progression from embryonic patterning to functional tissue formation and provides an essential framework for understanding how epigenetic dysregulation contributes to human developmental disorders and disease. Disruption of these processes can lead to profound physiological defects and human disease, underscoring the utility of zebrafish as a model for understanding epigenetic dysregulation in development and disease.

## 6. Zebrafish Models of Epigenetic Dysregulation in Human Disease

**Figure 4:**
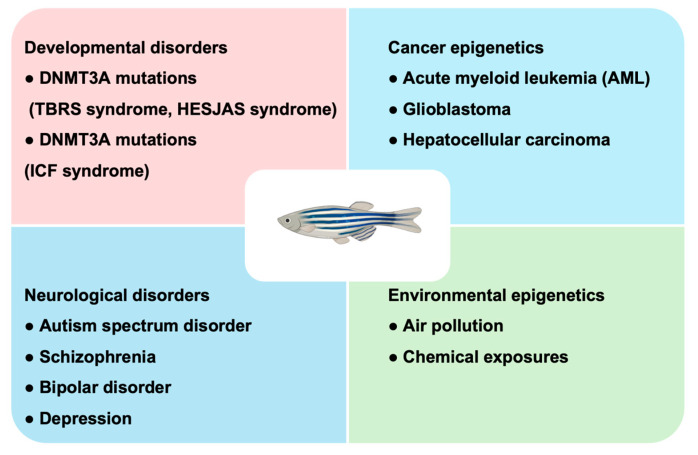
Zebrafish serve as a model to study DNMT3A-associated syndromes, cancer, neurological disorders, and environmental impacts on epigenetics.

The conservation of DNA methylation machinery and developmental programs between zebrafish and humans has enabled the use of zebrafish as a versatile vertebrate model for investigating diseases driven by epigenetic dysregulation. Disruption of *de novo* or maintenance methylation during development reshapes gene regulatory landscapes, leading to persistent defects in tissue organization, cellular function, and disease susceptibility. Zebrafish provide a tractable system for in vivo interrogation of these processes, from early embryogenesis through organogenesis and into adulthood (Summarized in Figure 4 and Table 2).

### 6.1. Developmental Disorders

Mutations in DNMT3A exemplify how perturbations of *de novo* methylation contribute to human disease. Germline DNMT3A mutations cause Tatton-Brown–Rahman (TBRS) syndrome, an overgrowth disorder with intellectual disability and variable neurodevelopmental abnormalities, and Heyn–Sproul–Jackson (HESJAS) syndrome, associated with microcephalic dwarfism [35,115]. Structural analyses suggest that TBRS-associated mutations disrupt interdomain interactions within DNMT3A and impair histone binding, thereby affecting *de novo* DNA methylation [35]. Disease-causing gain-of-function mutations, altering the PWWP domain mis localize DNMT3A, leading to DNA hypermethylation at DNA methylation valleys [115]. Mutations in DNMT1 have also been associated with adult-onset neurodegenerative disorders, including hereditary sensory and autonomic neuropathy with dementia and hearing loss. These mutations impair maintenance DNA methylation and lead to progressive neuronal dysfunction [119,120]. Zebrafish orthologs Dnmt3aa and Dnmt3ab recapitulate these phenotypes: both loss- and gain-of-function manipulations disrupt developmental gene generation, neural patterning, and tissue morphogenesis [121].

Centromeres are highly repetitive chromosomal regions defined by specialized epigenetic features that assemble kinetochores and ensure accurate chromosome segregation during mitosis and meiosis [122]. Proper epigenetic maintenance of these regions relies in part on DNMT3B-dependent DNA methylation, which helps preserve pericentromeric heterochromatin and genome stability [123,124]. In humans, loss-of-function mutations in *DNMT3B* cause immunodeficiency-centromeric instability-facial anomalies (ICF) syndrome, characterized by pericentromeric hypomethylation, chromosomal instability, and developmental defects [125]. DNMT3B-dependent maintenance of pericentromeric methylation is complemented by interactions with centromeric proteins, and non-catalytic isoforms may recruit catalytic DNMT3A variants to reinforce chromatin stability [126,127,128]. Similar to human patients, zebrafish *dnmt3b*-deficient models faithfully recapitulate these molecular and developmental features, including pericentromeric hypomethylation, chromosomal instability, and hematopoiesis defects [39]. Importantly, hypomethylation of pericentromeric repeats can trigger aberrant transcription of repetitive elements and activation of innate immune pathways, including interferon responses, providing mechanistic insight into immune dysfunction observed in ICF patients [39].

Beyond developmental disorders, DNA methylation dysregulation is a hallmark of cancer, particularly in hematological malignancies.

### 6.2. Cancer Epigenetics

DNMT3A is commonly mutated in acute myeloid leukemia (AML) and in clonal hematopoiesis of indeterminate potential, leading to focal hypermethylation at CpG islands and DNA methylation valleys alongside global hypomethylation in partially methylated domains [45,116]. In renal cell carcinoma, loss of SETD2-dependent H3K36 methylation perturbs DNMT3 targeting, contributing to global methylation changes and altered regulation at DNA methylation valleys [129]. Similarly, gain-of-function mutations in isocitrate dehydrogenase (IDH) generate the oncometabolite 2-hydroxyglutarate (2-HG), inhibiting TET-mediated α-ketoglutarate-dependent demethylation and histone lysine demethylases in both glioblastomas and myeloid leukemias [130].

Zebrafish have been established as a robust in vivo model to investigate methylome dynamics during chemical carcinogenesis. Comprehensive CpG islands methylation maps of adult zebrafish liver and hepatocellular carcinoma (HCC) have facilitated these studies [131]. Liver-specific overexpression of *uhrf1* in transgenic fish induces global hypomethylation, genomic instability, and spontaneous hepatocellular carcinoma, demonstrating a direct link between maintenance methylation machinery and cancer development [117].

### 6.3. Neurological Diseases

In addition to cancer, Aberrant DNA methylation also contributes to a range of central nervous system disorders, including autism spectrum disorder, schizophrenia, bipolar disorder, and depression [6]. Elevated DNMT1 and DNMT3A expression in cortical interneurons of affected individuals suggests that dysregulated DNA methylation contributes to transcriptional abnormalities in the diseased brain [132]. Zebrafish models recapitulated relevant behavioral phenotypes, including social withdrawal and cognitive impairment. For instance, administration of L-methionine, a methyl donor, enhances global DNA methylation and elevates promoter methylation of schizophrenia-associated genes such as *GABRB2*, mirroring epigenetic alterations observed in postmortem brain tissue from patients with schizophrenia [133]. Together, these studies highlight the utility of zebrafish for linking early epigenetic perturbations to later neural and behavioral phenotypes.

### 6.4. Environmental Toxicant-Induced Methylation Changes

Environmental factors can also alter DNA methylation landscapes, impacting gene regulation and influencing disease susceptibility [134]. Zebrafish embryos are highly amenable to controlled chemical exposures, providing a straightforward and scalable platform to investigate the epigenetic consequences of environmental toxins [135,136]. Compounds such as 17α-ethinylestradiol and arsenic induce locus-specific methylation changes, illustrating the translational relevance of zebrafish for environmental epigenetics and risk assessment [118].

Together, zebrafish models offer a uniquely tractable vertebrate system to dissect the molecular and developmental consequences of DNA methylation dysregulation. Across diverse disease contexts, disruption of DNA methylation, whether through impaired *de novo* methylation, defective maintenance, or altered demethylation, reshapes gene regulatory landscapes and leads to persistent defects in tissue organization, cellular function, and disease susceptibility. By enabling precise manipulation of epigenetic regulators and in vivo monitoring of tissue-specific outcomes, these models bridge molecular mechanisms and organismal phenotypes, providing insight into the pathogenesis of developmental disorders, cancer, neurological disease, and environmentally induced pathologies, while serving as a platform for testing epigenetic therapeutic strategies.

## 7. Emerging Technologies and Future Directions

Rapid advances in epigenomic technologies are transforming how DNA methylation is studied in vivo (summarized in Table 3), and zebrafish are particularly well-positioned to benefit from these developments. DNA methylation profiling technologies can be broadly categorized by their resolution, including bulk, single-cell, and spatially resolved approaches, each offering distinct advantages and limitations depending on the biological question.

Bulk sequencing approaches provide high coverage and quantitative accuracy but lack cellular resolution, whereas single-cell methods enable dissection of cellular heterogeneity at the cost of reduced coverage and increased technical complexity. Representative methods include whole-genome bisulfite sequencing (WGBS), which enables base-resolution methylation mapping across the entire genome [137], reduced representation bisulfite sequencing (RRBS), which enriches for CpG-dense regions such as promoters and CpG islands [138], and immunoprecipitation-based approaches such as MeDIP-seq, which offer a cost-effective profiling methylated DNA [139]. These approaches are particularly well suited for generating high-confidence reference methylomes and for comparative analyses across developmental stages or experimental conditions. When combined with the precise temporal control afforded by zebrafish embryogenesis, these technologies allow detailed reconstruction of methylation trajectories from fertilization through organogenesis.

Single-cell epigenomic technologies represent a major conceptual advance by enabling the resolution of cellular heterogeneity and lineage-specific epigenetic states. Techniques such as single-cell bisulfite sequencing (scBS-seq) and single-cell nucleosome, methylation and transcription sequencing (scNMT-seq) have now enabled the dissection of cell-type-specific methylation states and lineage trajectories that were previously inaccessible [140,141]. Although these methods typically involve reduced coverage and increased technical complexity, they are uniquely powerful for reconstructing lineage trajectories and linking epigenetic states to cell fate decisions. In zebrafish, where embryonic development is highly synchronized and lineage transitions occur rapidly, single-cell methylome profiling provides a particularly powerful approach to directly link DNA methylation dynamics with cell fate decisions. Integration of single-cell methylation data with single-cell transcriptomics further facilitates correlation of epigenetic state with gene expression programs, offering mechanistic insight into how DNA methylation shapes developmental trajectories and disease-associated phenotypes.

Spatial epigenomics adds an additional layer of resolution by preserving tissue architecture while profiling epigenetic states [142]. Unlike bulk or single-cell sequencing, spatially resolved technologies enable mapping of epigenetic heterogeneity within developmental niches and disease microenvironments. For example, combining Cleavage Under Targets and Tagmentation (CUT & Tag) with Multiplexed Error-Robust Fluorescence In Situ Hybridization (MERFISH) allows spatial mapping of epigenomic landscapes within intact tissues [143]. Similarly, the Spatial-CUT & Tag, coupling chromatin profiling with next-generation sequencing, enables unbiased, genome-wide discovery of epigenomic features [144]. These approaches are particularly well suited for linking epigenetic regulation to tissue organization and microenvironmental context. Zebrafish are particularly advantageous for such spatial epigenomic studies owing to their optical transparency, external development, and rapid organogenesis. Recent work integrating in vivo transcriptomic and epigenomic profiling in zebrafish larvae has delineated cardiomyocyte-specific landscapes of candidate regulatory elements (cREs) that drive cardiac development [145]. As spatial multi-omics technologies advance, zebrafish is likely to become an important system for integrating spatial methylome profiling with transcriptional and chromatin analyses [90].

Beyond descriptive profiling, functional interrogation of DNA methylation pathways has also been accelerated by CRISPR-based technologies. The zebrafish system is particularly well-suited for large-scale genetic screens owing to its high fecundity and experimental accessibility. CRISPR/Cas9-based genetic screening approaches using multiplexed Cas9 ribonucleoprotein injections enable efficient mosaic mutagenesis and rapid phenotypic assessment without generating stable mutant lines. For example, multiplex CRISPR screening in zebrafish identified regulators of retinal pigment epithelium regeneration by targeting candidate genes and assessing regenerative phenotypes in vivo [146]. Such approaches can be combined with genome-wide DNA methylation profiling to identify genes that regulate methylation establishment, maintenance, and interpretation.

**Table 3:** Assays and readouts for methylation studies in zebrafish.

Assay	Primary Readout	Resolution	Sample Input	Advantages	Limitations	Typical Applications	Cost
WGBS [137]	Genome-wide CpG and non-CpG methylation	Single-base	High (ng–µg DNA)	Comprehensive, unbiased	High cost; deep sequencing required	Global methylome profiling; developmental dynamics	High
RRBS [138]	CpG-rich regions (promoters, CpG islands)	Single-base	Moderate	Cost-effective; high coverage of regulatory regions	Biased toward CpG-dense regions	Comparative methylation studies	Medium
MeDIP-seq [139]	Methylated DNA enrichment	~100–300 bp	Low–moderate	Low input; scalable	Low resolution; antibody bias	Global methylation trends	Medium
Bisulfite PCR/amplicon BS-seq [147]	Locus-specific methylation	Single-base	Very low	High sensitivity; inexpensive	Limited to selected loci	Validation of DMRs	Low
CUT & Tag (5mC/5hmC) [144]	Targeted methylation-associated chromatin	~100 bp	Very low (single embryos)	Low input; cell-type specificity	Antibody-dependent; limited targets	Tissue- or cell-type–specific profiling	Medium
Single-cell methylome sequencing [140]	Cell-resolved DNA methylation	Single-cell	Single cells	Resolves heterogeneity; lineage inference	Technically challenging; sparse data	Developmental lineage analysis	High
RNA-seq [90]	Gene expression	Gene-level	Low–moderate	Functional consequence of methylation	Indirect readout	Methylation–expression integration	Medium
ATAC-seq [148]	Chromatin accessibility	~50 bp	Low	Links methylation to chromatin state	Does not measure methylation directly	Regulatory element analysis	Medium
Imaging-based reporters [149]	Tissue/organ morphology	Cellular	Whole embryos	Real-time, spatial context	No direct methylation readout	Developmental phenotyping	Low
Behavioral assays [150]	Functional output	Organismal	Larvae/adults	Disease-relevant phenotypes	Indirect; multifactorial	Neurodevelopmental studies	Low

In addition, CRISPR-based epigenome editing further expands the experimental toolkit. In zebrafish, targeted DNA methylation editing has been developed by fusing catalytically inactive Cas9 (dCas9) to the catalytic domains of zebrafish Dnmt3ba or Tet2. These systems enable locus-specific methylation or demethylation at selected promoters, including dmrt1 and cyp19a1a [121]. Targeted methylation mediated by dCas9-Dnmt3baCD can be detectable as early as 24 h post-fertilization, whereas dCas9-Tet2CD induces more modest demethylation, likely reflecting developmental constraints on Tet activity during early embryogenesis [121]. Such strategies offer a powerful framework for directly testing causal relationships between methylation changes at defined regulatory elements and developmental or disease phenotypes.

Increasingly, DNA methylation is being studied as part of an integrated epigenetic landscape that includes chromatin accessibility, histone modifications, and transcriptional regulation. Integrative multi-omics approaches in zebrafish have revealed how these regulatory layers interact during development [149]. For example, WGBS and RNA-seq analyses during early embryogenesis have demonstrated coordinated methylome remodeling and activation of zygotic gene expression programs [151]. Parallel ATAC-seq profiling has uncovered dynamic waves of chromatin opening at promoters and enhancers associated with lineage specification and organogenesis [149]. Notably, enhancer demethylation frequently aligns with increased chromatin accessibility and transcriptional activation, whereas promoter methylation alone is not always sufficient to predict transcriptional repression [152]. Single-cell multi-omics analyses of zebrafish germ cells further reveal stage-specific methylation remodeling and chromatin compaction during spermatogenesis, with a subset of regulatory loci remaining accessible and potentially contributing to intergenerational epigenetic inheritance [152].

Despite rapid progress, important questions remain. How DNMT3 activity is regulated in vertebrate lineages lacking canonical cofactors such as DNMT3L, how non-catalytic DNMT3 paralogs function as regulatory scaffolds in vivo, and how early epigenetic perturbations propagate to produce late-onset disease phenotypes remain largely unresolved. Addressing these questions will require continued integration of emerging technologies with powerful developmental systems.

Zebrafish represent a uniquely versatile platform for future epigenetic research. Their experimental accessibility, conserved epigenetic machinery, and compatibility with advanced genomic and imaging technologies position zebrafish at the forefront of efforts to understand DNA methylation in development and disease. As these technologies continue to evolve, zebrafish-based studies are poised to yield fundamental insights into DNA methylation regulation and accelerate the translation of epigenetic knowledge into therapeutic and environmental health applications.

## 8. Conclusions

DNA methylation represents a central regulatory mechanism that integrates developmental history with long-term control of gene expression, and its dysregulation contributes to a wide spectrum of human diseases [153]. Studies in zebrafish have revealed that, despite clear differences in early embryonic strategies compared with mammals, core principles governing DNA methylation are deeply conserved across vertebrates [3]. Zebrafish therefore provide a powerful and complementary perspective for understanding how DNA methylation is established, interpreted, and perturbed during development.

One of the major strengths of zebrafish lies in the combination of experimental accessibility and vertebrate relevance [21]. External fertilization, optical transparency, and rapid embryonic development enable direct interrogation of epigenetic processes at stages that are difficult to access in mammalian systems. High fecundity further supports large-scale genetic and chemical screens, facilitating systematic and quantitative analysis of methylation-dependent phenotypes in vivo. At the molecular level, the core epigenetic machinery, including DNMT and TET enzymes, is evolutionarily conserved, and genome-wide methylation profiling demonstrates broad architectural similarity between zebrafish and mammalian methylomes. Together, these features have established zebrafish as an indispensable vertebrate system for dissecting the temporal and spatial logic of DNA methylation regulation.

At the same time, important biological differences between zebrafish and mammals provide valuable comparative insights into epigenetic regulation. Zebrafish development does not rely on canonical genomic imprinting for viability and exhibits limited genomic imprinting compared to mammals. Early embryonic methylation reprogramming also differs from that observed in mammals, including stable inheritance of the paternal methylome after fertilization. Zebrafish further lacks a clear ortholog of *DNMT3L*, a critical regulator of germline methylation in mammals, suggesting that alternative regulatory mechanisms can support robust *de novo* methylation and development progression. These differences reflect broader species-specific regulatory features that may influence the interpretation of epigenetic studies. For example, the absence of imprinting and extraembryonic tissues in zebrafish reduces the requirement for genome-wide epigenetic reprogramming, thereby altering the dynamics and functional context of DNA methylation during early development. In addition, although many core components of the DNA methylation machinery are conserved, some cis-regulatory elements and noncoding genomic features are not fully conserved between fish and humans, potentially limiting direct extrapolation of locus-specific findings [69].

Beyond mechanistic studies, zebrafish models have provided important links between developmental DNA methylation and human disease. Functional analyses of DNMT-associated disorders demonstrate how early perturbations in DNA methylation dynamics can propagate through development to produce pathological phenotypes. Moreover, the ability to manipulate DNA methylation regulators in vivo and to observe the resulting effects across cellular, tissue, and organismal levels makes zebrafish a powerful system for linking molecular mechanisms to disease etiology. Meanwhile, several limitations should be considered when translating findings from zebrafish to human disease. Differences in physiology, tissue complexity, and lifespan, as well as species-specific regulatory architectures, may influence the extent to which zebrafish phenotypes recapitulate human pathology. Accordingly, although zebrafish provide a powerful and experimentally accessible platform for studying DNA methylation–mediated disease mechanisms, careful integration with mammalian models is essential to ensure accurate translation to human biology.

DNA methylation has also emerged as an important therapeutic target. The clinical success of DNMT inhibitors such as azacitidine [154] and decitabine [155] in the treatment of myelodysplastic syndrome and related hematologic malignancies highlights the translational relevance of epigenetic regulation. However, these first-generation DNMT inhibitors act broadly and lack locus specificity, underscoring the need for next-generation epigenetic therapeutics with improved precision and reduced toxicity. In this context, zebrafish offer a powerful vertebrate platform for DNA methylation-targeted drug discovery. Moreover, zebrafish disease models carrying mutations in DNMTs, TETs, or methylation-sensitive regulatory circuits allow functional validation of candidate compounds. In addition, zebrafish provide a scalable system for investigating how environmental exposures reshape the methylome and interact with pharmacological interventions, expanding their utility in environmental epigenetics and therapeutic development.

**Figure 5:**
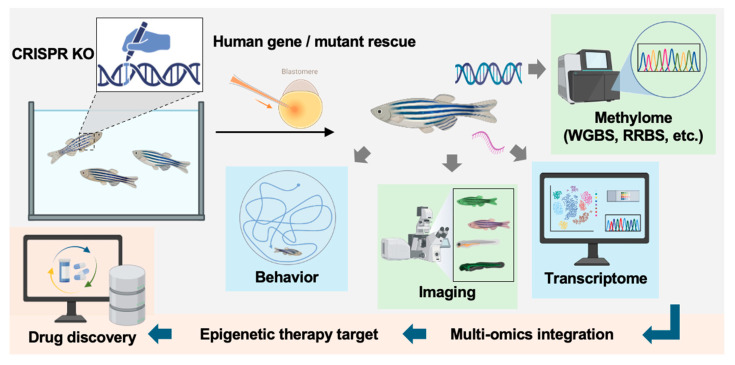
Zebrafish provide a powerful platform for bridging human disease biology and therapeutic discovery. Schematic overview of the experimental workflow. CRISPR knockout (KO) is used to generate loss-of-function models, followed by transgenic rescue with human DNMT3 wild-type (WT) or disease-associated variants. Resulting phenotypes are assessed at multiple levels, including behavioral analysis, imaging-based characterization, and molecular profiling. Multi-omics approaches, such as methylome analysis (e.g., WGBS, RRBS) and transcriptome sequencing, are integrated to elucidate gene function and regulatory mechanisms. These data collectively inform epigenetic target identification and support downstream drug discovery efforts. Created in BioRender. Xu, T. (2026) https://BioRender.com/fzwtoiz. Accessed on 28 April 2026.

Looking forward, the integration of emerging technologies promises to further expand the impact of zebrafish in epigenetic research. Advances in single-cell and spatial epigenomics, CRISPR-based epigenome editing, and integrative multi-omics approaches will enable increasingly precise dissection of DNA methylation dynamics in vivo. In particular, emerging long-read sequencing technologies offer new opportunities to resolve DNA methylation patterns at single-molecule resolution across repetitive and structurally complex genomic regions that are difficult to assess using short-read approaches. By preserving long-range genomic context, these platforms enable phasing of DNA methylation with genetic variants and chromatin features, thereby providing a more comprehensive view of epigenetic regulation and facilitating analysis of allele-specific methylation and epigenetic heterogeneity. In parallel, stage-specific and cell-type-resolved analyses will be essential for understanding how dynamic DNA methylation changes guide developmental transitions and contribute to disease susceptibility. By bridging conserved molecular mechanisms with organism-level phenotypes, zebrafish studies will continue to refine our understanding of epigenetic regulation and accelerate the translation of epigenetic knowledge into therapeutic and environmental health applications (summarized in Figure 5).

## Figures and Tables

**Table 1:** Zebrafish DNA methylation enzymes and proposed correspondence to human DNMT/TET factors.

Zebrafish Gene	Human Counterpart	Relationship	Predicted Activity	Key Domains/Features	Developmental Expression & Function	Disease Modeling Relevance
*dnmt1*	*DNMT1*	Ortholog	Maintenance methyltransferase	RFTS, CXXC, BAH, MTase	Ubiquitous; required for maintenance of CpG methylation, genome stability, embryonic viability [32]	Global hypomethylation models; genome instability; cancer-related methylation defects [33]
*dnmt2*	*DNMT2*	Ortholog	tRNA methyltransferase	MTase		
*dnmt3aa/* *dnmt8*	*DNMT3A* (*DNMT3A2*-like)	Functional ortholog	*De novo* methyltransferase	PWWP, ADD, MTase	Enriched in early embryogenesis; maternal and early zygotic expression [34]; methylome remodeling [33]	Modeling early DNMT3A dysfunction; developmental disorders (e.g., TBRS) [35]
*dnmt3ab/* *dnmt6*	*DNMT3A* (*DNMT3A1*-like)	Functional ortholog	*De novo* methyltransferase	PWWP, ADD, MTase	Broad expression during later development and differentiation [36]	DNMT3A-related neurodevelopmental disorders [37]; cancer-associated mutations [33]
*dnmt3ba/* *dnmt7*	*DNMT3B*	Ortholog	*De novo* methyltransferase	PWWP, ADD, MTase	Expressed during development; contributes to chromatin regulation and genome stability [38]	DNMT3B-associated developmental defects; ICF-like mechanisms [39]
*dnmt3bb.1/* *dnmt4*	*DNMT3B*	Ortholog	*De novo* methyltransferase	PWWP, ADD, MTase	Tissue-enriched (e.g., hematopoietic lineages); role in differentiation [38]	Hematopoiesis; leukemia and cancer epigenetics [40]
*dnmt3bb.2/* *dnmt3*	*DNMT3B*	Functional analog	*De novo* methyltransferase	PWWP, ADD; disrupted or inactive MTase	Expressed during development; predicted regulatory role in DNMT3 complexes [38]	Modeling non-catalytic DNMT3 regulation; structure–function studies [33]
*dnmt3bb.3/* *dnmt5*	*DNMT3B*	Functional analog	*De novo* methyltransferase	PWWP, ADD, MTase	Expressed in brain; digestive system; musculature system; and pleuroperitoneal region [41]	ICF-like mechanisms; autoimmune disease; carcinoma; thymoma [42]
-	*DNMT3L*	Absent	Regulatory cofactor (mammals only)	CD-like domain; no MTase activity	Not present in zebrafish; regulatory functions likely compensated by DNMT3 paralogs [21]	Reveals alternative regulatory strategies for *de novo* methylation [31]
*tet1*	*TET1*	Ortholog	5mC oxidation (demethylation)	CXXC, DSBH catalytic domain	Early development; dynamic methylation remodeling [43]	Epigenetic plasticity; developmental regulation [44]
*tet2*	*TET2*	Ortholog	5mC oxidation (demethylation)	DSBH catalytic domain	Hematopoiesis and tissue differentiation [45]	Hematologic malignancies; clonal disorders [45]
*tet3*	*TET3*	Ortholog	5mC oxidation (demethylation)	CXXC, DSBH catalytic domain	Early embryogenesis; epigenetic reprogramming [3]	Early developmental epigenetic regulation [3]

**Table 2:** Summary of zebrafish models used to investigate human diseases driven by epigenetic dysregulation.

Disease Type	Key Methylation Change	Zebrafish Model	Key Findings	Relevance to Human Disease
Developmental disorders (TBRS, HESJAS) [35,115]	Aberrant *de novo* methylation, DMV hypermethylation	*dnmt3aa*/*dnmt3ab* LOF/GOF	Neural defects, abnormal morphogenesis	Recapitulates DNMT3A mutation phenotypes
ICF syndrome [39]	Pericentromeric hypomethylation	*dnmt3b* mutants	Chromosomal instability, immune activation	Mirrors human genome instability
AML [45,116]	CpG island hypermethylation + global hypomethylation	*dnmt* perturbation models	Disrupted hematopoiesis	Models epigenetic drivers of leukemia
Liver cancer [117]	Global hypomethylation	*uhrf1* overexpression	HCC development, genomic instability	Links maintenance methylation to tumorigenesis
Environmental exposure [118]	Locus-specific methylation changes	Toxicant exposure	Epigenetic reprogramming	Models environmental epigenetics

## Data Availability

No new data were created or analyzed in this study.

## Abbreviations

The following abbreviations are used in this manuscript:

DNMT	DNA methyltransferase
TET	ten-eleven translocation proteins
CGIs	CpG islands
UHRF1	Ubiquitin-like with PHD and RING finger domains 1
BAH	The Bromo-Adjacent Homology domain
PWWP	Pro-Trp-Trp-Pro domain
ADD	the ATRX-DNMT3A-DNMT3L zinc-finger domain
MTase	DNA methyltransferase
cryo-EM	Cryogenic electron microscopy
5mC	5-methylcytosine
5hmC	5-hydroxymethylcytosine
5fC	5-formylcytosine
5caC	5-carboxycytosine
TDG	thymine DNA glycosylase
BER	base excision repair
PGCs	primordial germ cells
ICM	inner cell mass
DMV	DNA methylation valleys
MBT	midblastula transition
ZGA	zygotic genome activation
MZT	maternal-to-zygotic transition
HSC	hematopoietic stem cell
EMPs	erythromyeloid progenitors
AML	acute myeloid leukemia
MECP2	Methyl-CpG-binding protein 2
TBRS	Tatton-Brown–Rahman syndrome
HESJAS	Heyn–Sproul–Jackson syndrome
ICF	immunodeficiency-centromeric instability-facial anomalies syndrome
SETD2	SET domain-containing 2
IDH	isocitrate dehydrogenase
2-HG	2-hydroxyglutarate
HCC	hepatocellular carcinoma
WGBS	Whole-genome bisulfite sequencing
RRBS	reduced representation bisulfite sequencing
scBS-seq	single-cell bisulfite sequencing
scNMT-seq	single-cell nucleosome, methylation and transcription sequencing
CUT & Tag	Cleavage Under Targets and Tagmentation
MERFISH	Multiplexed Error-Robust Fluorescence In Situ Hybridization
cREs	candidate regulatory elements
ICR	Imprinting Control Region
TTD	Tudor domain
PHD	plant homeodomain
hpf	hours post-fertilization
